# Kinematic Chains in Ski Jumping In-run Posture

**DOI:** 10.2478/hukin-2013-0069

**Published:** 2013-12-31

**Authors:** Eva Janurová, Miroslav Janura, Lee Cabell, Zdeněk Svoboda, Ivan Vařeka, Milan Elfmark

**Affiliations:** 1 Institute of Physics, Faculty of Mining and Geology, Technical University of Ostrava, Czech Republic.; 2 Department of Natural Sciences in Kinanthropology, Faculty of Physical Culture, Palacky University, Olomouc, Czech Republic.; 3 Department of Graduate Programs in Health Sciences, School of Graduate Medical Education, Seton Hall University, South Orange, New Jersey, U.S.A.; 4 Department of Physiotherapy, Faculty of Physical Culture, Palacky University, Olomouc, Czech Republic.

**Keywords:** 2-D videography, biomechanics, ski jumping, kinematic chains

## Abstract

The concept of kinematic chains has been systematically applied to biological systems since the 1950s. The course of a ski jump can be characterized as a change between closed and open kinematic chains. The purpose of this study was to determine a relationship between adjacent segments within the ski jumper’s body’s kinematic chain during the in-run phase of the ski jump. The in-run positions of 267 elite male ski jumpers who participated in the FIS World Cup events in Innsbruck, Austria, between 1992 and 2001 were analyzed (656 jumps). Two-dimensional (2-D) kinematic data were collected from the bodies of the subjects. Relationships between adjacent segments of the kinematic chain in the ski jumper’s body at the in-run position are greater nearer the chain’s ground contact. The coefficient of determination between the ankle and knee joint angles is 0.67. Changes in the segments’ positions in the kinematic chain of the ski jumper’s body are stable during longitudinal assessment. Changes in shank and thigh positions, in the sense of increase or decrease, are the same.

## Introduction

Kinematic chains have been studied since the 1870s (Reuleaux, 1875). Di Fabio (1999) states that these first works to study kinematic chains in industrial conditions described the structure of various single, rigid co mponents or segments linked together by a pin. More recently, the concept of kinematic chains has been applied to biological systems of movement.

A linkage of rigid bodies can be referred as a kinetic chain (Zatsiorsky, 1997). If an exertion of external and internal forces is added to the system of segments, it is referred to as a “kinetic chain.” Steindler (1955) used the term “kinetic chain” to describe the movement of body segments.

Kinetic chains are divided into two categories – open (OKC) and closed (CKC) – based on the method of setting the segments and their fixation(s) during movement. Exercises with OKCs involve unrestrained movement of a limb’s distal joint, more easily isolating muscle groups, while CKC exercises have constraints on both ends (Zatsiorsky, 1997). The application of the kinetic chain is now widely used in sport biomechanics (Stepien et al., 2011) and more recently in physical therapy, where it can aid in the choice of appropriate exercises for rehabilitation and training (Escamilla et al., 1998; Graham et al., 1993).

The movements of living systems are typically very complicated. Therefore, a simplification of entry factors is necessary. Depicting the human body as a set of rigid parts linked in a kinetic chain is a frequently-applied procedure (Sundaresan et al., 2004), and this biomechanical construct should be sufficiently sophisticated to represent the human body realistically (Zhang, 2001).

An example of this application is the study of a ski jumper’s in-run, when his positions in a sagittal plane are significantly influenced by the skis’ alignment in the in-run track. The in-run body position is designed to achieve optimal (maximum) in-run velocity, whereby the positions of body segments are of key importance in order to achieve maximum jump length (Dželalija et al., 2003). Individual body segments in the in-run position influence take-off execution, thus, it is already necessary to give attention to it in the process of “dry-land” exercises (Schwameder et al., 1997).

The purpose of this study was to determine a relationship between adjacent segments within the ski jumper’s body’s kinematic chain during the inrun, and to examine longitudinal changes in the inrun positions of body segments of selected jumpers.

## Material and Methods

### Participants

Elite male ski jumpers (N = 267) participated in the FIS World Cup events on the K110m ski jumping hill in Innsbruck, Austria, between 1992 and 2001. We analyzed a total of 656 jumps from this period. Average values were established based on angle parameters from competitors who had performed more than one jump during this period. Of all the subjects, we chose three top athletes who had competed frequently during the study period (over a 10-year period: athlete A – 10 jumps; athletes B and C – 9 jumps each).

### Measure

During the competitions, 2D video image data were recorded using one stationary camera (either a Grundig S-VHS 180 [1992–1999] or a Sony DCR-TRV 900 [2000–2001]) with a sampling frequency of 50 Hz. All images were registered from the same location, perpendicular to the ski track along the initial straight part of the inrun, 18 m from the edge of the jumping hill.

### Analysis

A six-link bilateral model was developed based on nine points on the ski jumper’s body and Centre of Mass (COM) according to Dempster (1955). The model included the following segments: foot, shank, thigh, trunk+head+neck, upper arm, and forearm (hand included). All joints had a single degree of freedom, with lumped inertia, damping, and stiffness. Each segment was composed of an ideal, rigid part.

**Figure 1:**
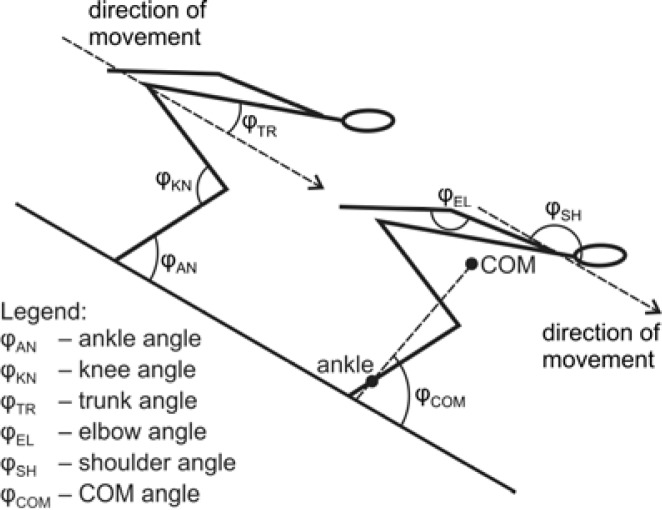
Body segmental angles. Note: The COM angle φ_COM_ is defined as the angle between the line connecting the body’s COM and ankle and the tangential component of movement.

We calculated the body’s COM by the relative weight of each segment and included the weight of gear (ski suit and helmet). Kinematic data were collected from the ankle, knee, hip, shoulder, elbow, and the body COM in the sagittal plane (Figure 1). Data were analyzed by software written in Pascal (Vaverka et al., 1994). A single person evaluated all records. Given the image resolution, each shift of the cursor by one pixel represented 0.003 m. The accuracy of angle values was quantified in previous studies (Janura and Vaverka, 1997). While the relative error value in the laboratory condition was 0.51% and absolute error was 0.22°, the maximum difference between real angles under the field conditions and analyzed angles did not exceed the value of 3°. Statistical analysis (Pearson coefficient, partial correlation) was performed using STATISTICA v9.0 (Stat-Soft, Inc., Tulsa, OK, USA). The level of significance was set at p<0.05.

## Results

The descriptive characteristics of the measured in-run position angle parameters for the group of 267 ski jumpers are given in Table 1.

**Figure 2:**
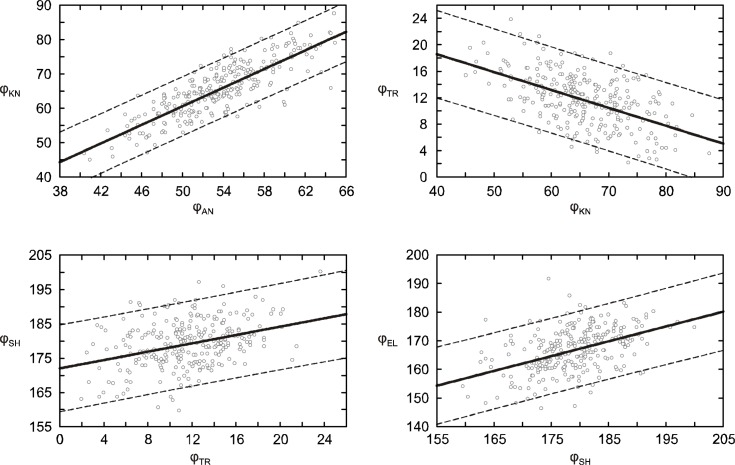
The relationship between angles (measured in degrees) of adjacent joints of the kinematic chain for a ski jumper’s in-run posture

The coefficient of determination between the ankle (φ_AN_) and knee (φ_KN_) joint angles is 0.67 and 0.34 between the knee joint (φ_KN_) and trunk (φ_TR_) angles. For the upper-limb distal segments (φ_SH_, φ_EL_), which are the free ends of the kinematic chain, the coefficient of determination is 0.29. With the exception of the relationship between changes in the knee and hip joints (where an increase in the angle of one results in a decrease in the angle of the other), changes in neighboring joints tend to have the same orientation (Figure 2). The COM is most influenced by the shank and the trunk position. The coefficients of partial correlation equal 0.74 for the ankle joint and 0.27 for the hip joint, respectively.

**Figure 3:**
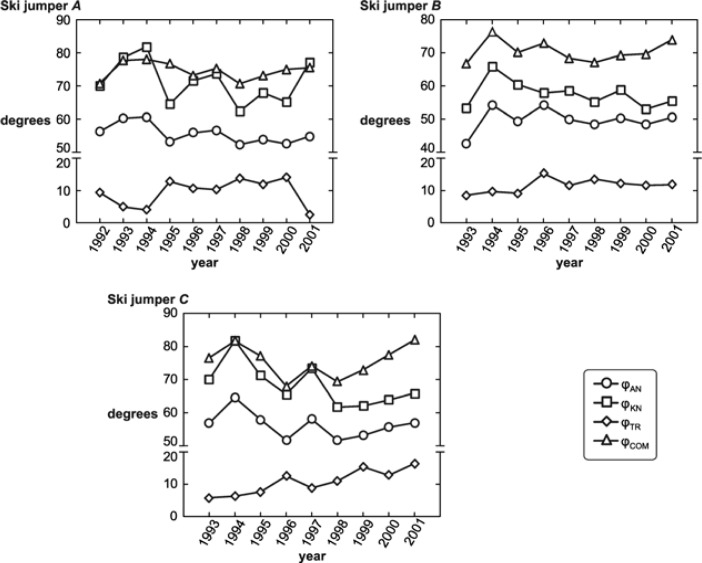
Graphic depiction of angles φ_AN_, φ_KN_, φ_TR_ and φ_COM_ for jumpers A (over 10 years), B (over 9 years), and C (over 9 years)

Figure 3 depicts the longitudinal monitoring of change in measured angles of three jumpers (A, B, C) who had competed at least nine times over the 10-year monitoring period.

Jumper A: The trends in changes of angles φ_AN_ and φ_KN_ are the same throughout the monitored period for this athlete. Changes in φ_COM_ compared to angles φ_AN_ and φ_KN_ differ in 1996 and 2000. The trend in changes of trunk posture is opposite to changes of angles φ_AN_ and φ_KN_ throughout the entire period.

Jumper B: With the exception of 1996, the trend of changes in angles φ_AN_ and φ_KN_ is the same across all years for this athlete. Changes in the angle φ_COM_ are the same as φ_AN_, with the exception of year 2000. Differences in the angle φ_TR_ are small over the monitored period.

Jumper C: The trend in changes of angles φ_AN_, φ_KN_, and φ_COM_ is consistent throughout the entire monitored period for this athlete. A change in the trunk posture has a tendency to increase the size of the angle (φ_TR_).

## Discussion

Understanding of the human body and its segmental chains is based on understanding of anatomical characteristics. It should be noted that the biological conditions of the human body must be considered in relation to the action of the kinematic chain (Tsarouchas, 1985). In such cases, the use of either open or closed chains can generate significantly different results, even if there are only minor changes in segment positions. Steindler (1977) explained that velocity and acceleration are more important for OKC exercises, while strength is more important for CKC exercises.

From a neurophysiological perspective, an OKC is characterized by the activity of one muscle or muscle group operating on a single joint. The activity of a CKC is represented by multi-joint movements with controlled muscle co-contractions (Di Fabio, 1999). For this reason, most regular movements or activities can be classified as a CKC, and include a ski jumper’s posture during the inrun, as examined in this study.

One of a ski jumper’s primary challenges is to maintain stability during the in-run, and to react to external changes, such as the wind and the quality of the in-run track. Because a jumper’s boots prevent plantar flexion during take-off, the knee joints play a primary role in the body’s segment chains (Virmavirta and Komi, 2001). For the in-run posture, knee joints react to changes in external conditions in order to maintain balance. The knee, therefore, is the most important joint used to generate acceleration until take-off and could be considered the primary drive system of the lower extremities’ biokinematic chain (Tsarouchas, 1985).

In our study, the in-run posture of a ski jumper is modeled from individual segments (with the exception of the hands) in which the paired segments of the upper and lower limbs are always replaced by a single segment having double the weight. Although the body’s movement during an in-run is not planar, the body can be simplified and replaced by a set of segments of which movements are along a sagittal plane.

During an in-run, the ski jumper focuses on maximizing in-run speed, minimizing friction between the skis and snow, and minimizing aerodynamic drag (Müller, 2005). During the initial flat section of the in-run, the jumper aims for a posture that will achieve an optimum take-off without loss of velocity, while maintaining a stable position by modifying muscle activity to make small changes in hip, knee, and ankle movements (Janura et al., 2010). Such changes lead to an anterior shift in the Centre of Pressure (COP) (Ettema et al., 2005).

Contrary to sports disciplines in which the athlete utilizes the rebound of sports shoes, the ski jumper’s primary in-run posture focus for the ankle joint is to optimize the torque and balance of the link drive system. Changes in the in-run body positions reflect the body posture during take-off (Zanevskyy and Banakh, 2010). Positional changes in individual segments affect the activation patterns of specific muscles which coordinate the body segments during take-off (Sasaki et al., 1993).

The ski jumper’s body takes the form of a “nonstationary” CKC while the feet are in contact with the ramp. The joint angular motions are coupled. When determining relationships and linkages for the position of individual segments, it is apparent that the closest dependence is in the framework of a six-link bilateral chain of adjoining segments. Segments at the end of the chain have lower mass, as they must be moved by a larger degree in order to affect the body’s movement as a whole (Hudson, 1986).

Longitudinal monitoring of selected jumpers has revealed a close relationship between changes in the position of adjacent segments of lower extremities (shanks and thighs). In most cases, the COM copies position changes of lower extremities. The relationship between changes in the trunk’s position and COM is not high. The complexity of movement structures is apparent in the inter-individual variability when executing the individual stages of a jump, not only among individual groups of jumpers with different performance levels, but also within a particular performance level group (Janura et al., 2007; Virmavirta et al., 2005; Vodičar et al., 2012). When applying results of the present study to training exercises, one must be aware that changes in the inrun posture mean that changes in the angle of one segment will automatically change the angles of adjoining segments.

### Limits of the study

Several factors may have influenced the posture of jumpers observed over the 10-year period: (1) changes in anthropometric parameters (body mass); (2) changes of in-run velocity; (3) progress in clothing and equipment material; (4) changes in flight techniques; (5) changes in jumping hill’s inrun profile; and (6) different weather conditions.

The amount of muscle mass in a particular body position limits the range of motion, specifically in the knee joint (Vaverka, 1987). Jumping hill improvement increased in-run velocities and decreased demand on postural stability. These variables can be considered as limitations of the study. However, these limits increase the validity of obtained data paradoxically in that the trend in dependence of changes in the angles applies throughout the entire observed period.

## Figures and Tables

**Table 1:** Basic characteristics of the in-run position angle parameters (Innsbruck, K110m, 1992–2001)

N=267	Mean	SD	Median	Minimum	Maximum
LJ [m]	90.11	9.47	91.06	58.00	111.50
v [km/h]	89.50	0.86	89.50	87.20	91.52
φ_AN_ [°]	54.06	4.65	54.06	35.33	68.78
φ_KN_ [°]	66.86	7.51	66.69	45.91	90.38
φ_TR_ [°]	11.59	3.62	11.57	3.07	22.76
φ_SH_ [°]	179.45	5.64	179.48	161.04	195.10
φ_EL_ [°]	165.80	7.03	166.81	141.80	179.96
φ_COM_ [°]	73.94	3.82	74.05	61.42	86.16

*LJ – length of jump, v – in-run velocity,* φ*_AN_ – ankle angle,* φ*_KN_ – knee angle,* φ*_TR_ – trunk angle,* φ*_SH_ – shoulder angle,* φ*_EL_ – elbow angle,* φ*_COM_ – COM angle (angle between COM and ankle connection and the tangential component of movement), SD – standard deviation*

## References

[b1-jhk-39-67] Dempster WT (1955). The space requirements of the seated operator [Technical Report No. WADC-TR-55-159].

[b2-jhk-39-67] Di Fabio RP (1999). Making jargon from kinetic and kinematic chains. J Orthop Sports Phys Ther.

[b3-jhk-39-67] Dželalija M, Rausavljevič N, Jošt B (2003). Relationship between jump length and the position angle in ski jumping. Kinesiol Slov.

[b4-jhk-39-67] Escamilla RF, Fleisig GS, Zheng N, Barrentine SW, Wilk KE, Andrews JR (1998). Biomechanics of the knee during closed kinetic chain and open kinetic chain exercises. Med Sci Sports Exerc.

[b5-jhk-39-67] Ettema GJC, Bråten S, Bobbert MF (2005). Dynamics of the in-run in ski jumping: A simulation study. J Appl Biomech.

[b6-jhk-39-67] Graham VL, Gehisen GM, Edwards JA (1993). Electromyographic evaluation of closed and open kinetic chain knee rehabilitation exercises. J Athl Training.

[b7-jhk-39-67] Hudson JL (1986). Coordination of segments in the vertical jump. Med Sci Sports Exerc.

[b8-jhk-39-67] Janura M, Cabell L, Elfmark M, Vaverka F (2010). Kinematic characteristics of the ski jump inrun: A 10-year longitudinal study. J Appl Biomech.

[b9-jhk-39-67] Janura M, Svoboda Z, Uhlář R, Linnamo V, Komi PV, Müller E (2007). A comparison of ski jump execution in a group of the best jumpers. Science and Nordic Skiing.

[b10-jhk-39-67] Janura M, Vaverka F (1997). Evaluation of the video analysis system I. (Precision of the analyzed data). Tel vých šport.

[b11-jhk-39-67] Müller W, Fleischer R (2005). The physics of ski jumping. Proceedings of European School of High-Energy Physics.

[b12-jhk-39-67] Reuleaux F (1875). Theoretical kinematics - outline of a theory of machines.

[b13-jhk-39-67] Sasaki T, Tsunoda K, Uchida E (1993). The effect of segment power in ski jumping. Proceedings of the 14th Congress of the International Society of Biomechanics in Sports.

[b14-jhk-39-67] Schwameder H, Müller E, Raschner C, Brunner F, Müller E, Schwameder H, Kornexl E, Raschner C (1997). Aspects of technique-specific strength training in ski jumping. Science and Skiing.

[b15-jhk-39-67] Steindler A (1955). Kinesiology of the human body under normal and pathological conditions.

[b16-jhk-39-67] Steindler A (1977). Kinesiology of the human body under normal and pathological conditions.

[b17-jhk-39-67] Stepien A, Bober T, Zawadzki J (2011). The kinematics of trunk and upper extremities in one-handed and two-handed backhand stroke. J Hum Kinet.

[b18-jhk-39-67] Sundaresan A, RoyChowdhury A, Chellappa R (2004). 3D modelling of human motion using kinematic chains and multiple cameras for tracking. Proceedings of the 8th International Symposium on the 3-D Analysis of Human Movement.

[b19-jhk-39-67] Tsarouchas E, Terauds J, Barham JN (1985). Optimization of the kinematic chain in human movement as it relates to training. Proceeding of the 3rd International Symposium on Biomechanics in Sports.

[b20-jhk-39-67] Vaverka F (1987). Biomechanics of ski jumping.

[b21-jhk-39-67] Vaverka F, Elfmark M, Janura M, Kršková M, Barabás A, Fabián G (1994). The system of kinematic analysis of ski-jumping. Proceedings of the 12th International Symposium on Biomechanics in Sports.

[b22-jhk-39-67] Virmavirta M, Isolehto J, Komi P, Brüggemann GP, Müller E, Schwameder H (2005). Characteristics of the early flight phase in the Olympic ski jumping competition. J Biomech.

[b23-jhk-39-67] Virmavirta M, Komi PV (2001). Plantar pressure and EMG activity of simulated and actual ski jumping take-off. Scand J Med Sci Sports.

[b24-jhk-39-67] Vodičar J, Čoh M, Jošt B (2012). The kinematics of trunk and upper extremities in one-handed and two-handed backhand stroke. J Hum Kinet.

[b25-jhk-39-67] Zanevskyy I, Banakh V (2010). Dependence of ski jump length on the skier’s body pose at the beginning of take-off. Acta of Bioengineering and Biomechanics.

[b26-jhk-39-67] Zatsiorsky VM (1997). Kinematics of human motion.

[b27-jhk-39-67] Zhang X (2001). Biomechanical realism versus algorithmic efficiency: a trade-off in human motion simulation and modeling. SAE Transaction.

